# Miscellany

**Published:** 1895-02

**Authors:** 


### Miscellany.

THE United States Department of Agriculture, through its Weather Bureau, is seeking to extend the scope of its work, as set forth in a circular recently issued. It has asked us to publish the text of the circular, which is as follows :

The interest manifested by every class of people in the subject of climate and its influence on health and disease has determined the Honorable the Secretary of Agriculture, through the medium of the Weather Bureau, to undertake the systematic investigation of the subject.

It is hoped to make the proposed investigation of interest and value to all, but especially to the medical and sanitary professions, and to the large number of persons who seek, by visitation of health resorts and change of climate, either to restore health or prolong lives incurably affected or to ward off threatened disease.

The study of the climates of the country in connection with the indigenous diseases should be of material service to every community, in showing to what degree local climatic peculiarities may favor or combat the development of the different diseases, and by suggesting, in many instances, supplementary sanitary precautions ; also by indicating to what parts of the country invalids and health seekers may be sent to find climatic surroundings best adapted to the alleviation or cure of their particular cases.

The hearty coöperation of the various boards of health, public sanitary authorities, sanitary associations and societies, and of physicians who may feel an interest in the work, is asked to achieve and perfect the aims of this investigation.

No compensation can be offered for this coöperation other than to send, free of cost, the publications of the Bureau bearing upon climatology and its relation to health and disease to all those who assist in the work.

Coöperation will consist in sending to this office reports of vital statistics from the various localities. That these reports may be of value, it is evident to all that they should be accurate and complete and be rendered promptly and regularly. Blank forms of reports have been prepared so as to occasion as little trouble and labor as possible on the part of the reporter, and will be furnished by the Bureau on application.

At the very beginning of the investigation it is not possible to outline precisely the channels through which the results obtained will be made public, but it is hoped to publish soon a periodical devoted to climatology and its relations to health and disease. The publication will probably resemble in size and general appearance the present *Monthly Weather Review*, the subject matter being, of course, different.

More detailed information will be furnished on application.

